# The Foodstuffs Advisory Board Specimen Menu

**Published:** 1917-06-16

**Authors:** 


### FOOD IN WARTIME.*

### The Foodstuffs Advisory Board Specimen Menu.

Much interest has been excited by the particulars lately given in these columns of the experiments conducted by the East Lancashire Branch of the Red Cross Society in victualling the military hospitals within their jurisdiction (see The Hospital, May 19, p. 136). It will be remembered that the committee discovered that where forty persons or more were boarded together on the lines they laid down, the average cost was no more than 1s. 8d. per head per diem. What did they give the men for this cost, with prices ruling at the rate of last March? In the first place they allowed for very ample quantities being consumed by the convalescent men for whom they catered. The weights allowed per person were as follows: 6 oz. meat for hot-pot, stew, potato pie, steak pudding, etc.; 8 oz. fish for dinner, ½ lb. for breakfast; 2 oz. liver, with 1½ oz. bacon for breakfast, or 3½ oz. of bacon grilled, or one new-laid egg; 2 oz. cold boiled ham for breakfast, or 2 oz. sausages; 8 oz. roast meat and stewed steak for dinner; one rabbit between three persons for dinner.

It is at once apparent that these quantities are in excess of those commonly allowed for in every-day life. Bread, vegetables, and puddings are given practically *ad lib.*

It is recommended that for breakfast, with tea and bread-and-butter daily, there should be bacon twice a week, sausage once, brawn and broth once, fresh fish or fish-cakes once, porridge once. But we prefer the alternative suggested of daily porridge with a small "follow" of the above dishes, as likely to give more satisfaction and lessen the consumption of the more expensive foods.

### A Week's Meals.

The dinner starts traditionally on *Sunday* with roast round of beef with suet pudding. *Monday*: Boiled leg of mutton, bread-and-butter pudding. *Tuesday*: Potato pie (made with what is left over from Sunday and Monday, with as much fresh meat as is required in addition), and rice pudding. *Wednesday*: Steak and kidney pie or pudding, or tripe, with baked jam-roll pudding. *Thursday*: Hot-pot (use necks or leg), with boiled pudding. *Friday*: Boiled cod, custard and rhubarb or stewed fruit. *Saturday*: Boiled brisket of beef, carrots, swedes, and some form of milk pudding. There is nothing very revolutionary in any of these days. But the caterer is entreated to make the main dish from each day help to supply the next day's wants. Broth obtained from the boiled mutton is to be utilised for breakfast or supper, fish-cakes are to be made from fish that is left over, and generally the idea is to provide occasions for using up the scraps.

A good suggestion is that during the summer months on one day a week corned beef should be provided for dinner. This can be purchased from dealers or from the B.R.C.S. at 80s. per case—*i.e.*, 1s. 1½d. per lb., cooked and without bone. The use of lentils, split peas, beans, rice, barley, etc., is recommended in soups or as vegetables to take the place of fresh ones; also that the monotony of the menu should be varied from time to time, and that as Wednesday stands by itself in this menu, it is a good day for introducing new dishes. Alternatives suggested for Tuesday are savoury hash, Winchester cutlets, lentil cutlets, meat fritters, rice cutlets, patties of cold meat, meat croquettes, rissoles of cold meat, minced beef and cheese, réchauffé of beef. Alternatives for Wednesday are steak and kidney pie or pudding, stewed tripe, beef roasted under potatoes, rabbit, stewed steak.

For tea, bread-and-butter, with jam or cake on alternate days; for supper, cocoa or milk, bread-and-butter, cheese, or broth are the recommendations.

Potatoes are to be boiled in their skins, unless peeled in a really good peeler, and what is left is to be used for hot-pot or sauté. For the moment, however, these are out of count. Too much attention cannot be given to the carving, which "is not a matter for the casual helper." But, alas! when will the art of carving be admitted as a fit subject for instruction to women who as sisters and matrons will certainly one day number it among their duties? The good housekeeper must often have the mortification of finding that well-chosen, well-cooked meat is hacked with a blunt knife into unappetising lumps by an unskilful hand, until the diner can scarce distinguish whether it be beef or mutton. With a really good knife, kept in condition by careful manipulation, with a strong wrist, and a pride in her craft, the carver is sure of success, and can not only lend a grace to each meal, but can also save many pounds in the course of the year.

### How to Serve Fish.

We are not altogether in accord with the above menu in all particulars. The Friday fish dinner is, in our judgment, too watery and unsubstantial for hungry convalescents, and, preceded as it is by haddock or herring for breakfast, is all the less likely to give satisfaction. Ordinary boiled fish is largely swelled out with water. Men are apt to swallow it without knowing they have had anything to eat, and feel almost as hungry afterwards as before. It could be served with boiled rice, lentils, or beans, or be stuffed and baked or broiled. In the form of fish pudding, flavoured with fried onions, or as kedgeree, fish is far more satisfying than plain boiled, and for making these savoury dishes any kind of the less expensive fish can be utilised. A crisp-baked treacle roll or baked raisin pudding would be more in keeping after a fish course than stewed fruit and custard, which supplies nothing "to bite on." Some men have a distaste for fish, and these should be provided with an alternative.

The danger of identifying any day of the week with a particular dish is commented on by those who frame the menu, and they urge that it should be avoided. The simplest way of doing so is to arrange for a menu of eight, nine, or ten days instead of seven. The tradition of Sunday roast beef could be preserved if desired. Another point worth mentioning is the liking for cold meat which is found among convalescent soldiers. When roast meat is left uncut to go cold all the juices are preserved in it, and it is not only very appetising but distinctly more nourishing, and therefore more economical, than when eaten hot. Boiled leg of mutton treated in this way is an excellent dish, and the addition of pickles is highly appreciated. If a little trouble were taken in the proper season to pickle cabbage, cauliflower, and onions—a process involving very little difficulty or labour—pickles would not be the expensive luxury they are at present. The craving for a "relish" universally displayed by the convalescent is a thing to be reckoned with.

* Previous articles appeared February 24, March 10, 24, and 31, April 14 and 21, May 5, 12, and 19, and June 9.

